# Conservative Management of Tibialis Anterior Muscle Herniation Prior to Surgical Intervention: A Case Report

**DOI:** 10.7759/cureus.110233

**Published:** 2026-06-04

**Authors:** Hajar Menay, Hajar Yazough, Manare Jaai, Ahmed Amine El Oumri

**Affiliations:** 1 Physical Medicine and Rehabilitation, Mohammed VI University Hospital, Faculty of Medicine and Pharmacy, Mohammed First University, Oujda, MAR; 2 Physical Medicine and Rehabilitation, Mohammed VI University of Health Sciences (UM6SS), Mohammed VI Foundation for Sciences and Health (FM6SS), Rabat, MAR

**Keywords:** conservative management, dynamic ultrasonography, fascial defect, muscle hernia, tibialis anterior muscle

## Abstract

Muscle herniation is a rare condition characterized by protrusion of muscle tissue through a fascial defect, most commonly involving the tibialis anterior muscle. We report the case of a 55-year-old man presenting with a painful intermittent swelling on the anterolateral aspect of the right leg that became more prominent during standing and physical activity following a remote history of trauma.

Physical examination revealed a soft, tender mass over the middle third of the leg without neurological deficits. Dynamic ultrasonography demonstrated a fascial defect with protrusion of the tibialis anterior muscle, confirming the diagnosis of tibialis anterior muscle herniation.

The patient was managed conservatively with rest and compression bandaging, resulting in significant pain relief and partial reduction in hernia size after a three-month follow-up.

This case highlights the importance of considering muscle herniation in the differential diagnosis of chronic leg pain and intermittent soft tissue swelling. Dynamic ultrasonography remains a valuable diagnostic tool, and conservative treatment may provide satisfactory outcomes in selected symptomatic patients.

## Introduction

Muscle herniation is defined as the protrusion of muscle tissue through a defect in the overlying fascia [1,2]. It is a rare condition that most commonly affects the tibialis anterior muscle due to its superficial location and increased intracompartmental pressure during physical activity [2,3]. Muscle hernias may be constitutional or acquired following traumatic events such as blunt trauma, repetitive overuse, or excessive muscular exertion [3,4]. Clinical presentation is variable and may include intermittent swelling, localized pain, soft tissue nodules, or cosmetic deformity that typically becomes more prominent during standing or muscle contraction [5].

Because of its variable presentation, tibialis anterior muscle herniation may mimic other soft tissue conditions, including lipoma, varicosities, vascular malformations, muscle rupture, or soft tissue tumors [5,6]. Dynamic ultrasonography is considered the imaging modality of choice, allowing real-time visualization of fascial defects and muscle protrusion, while magnetic resonance imaging may be useful in selected cases for surgical planning and evaluation of surrounding soft tissues [6,7].

Management options range from conservative treatment, including rest, activity modification, and compression therapy, to surgical intervention in symptomatic or refractory cases [7,8].

This case report aims to highlight the diagnostic value of dynamic ultrasonography and the potential effectiveness of conservative management in a patient with symptomatic tibialis anterior muscle herniation.

## Case presentation

**Figure 1:**
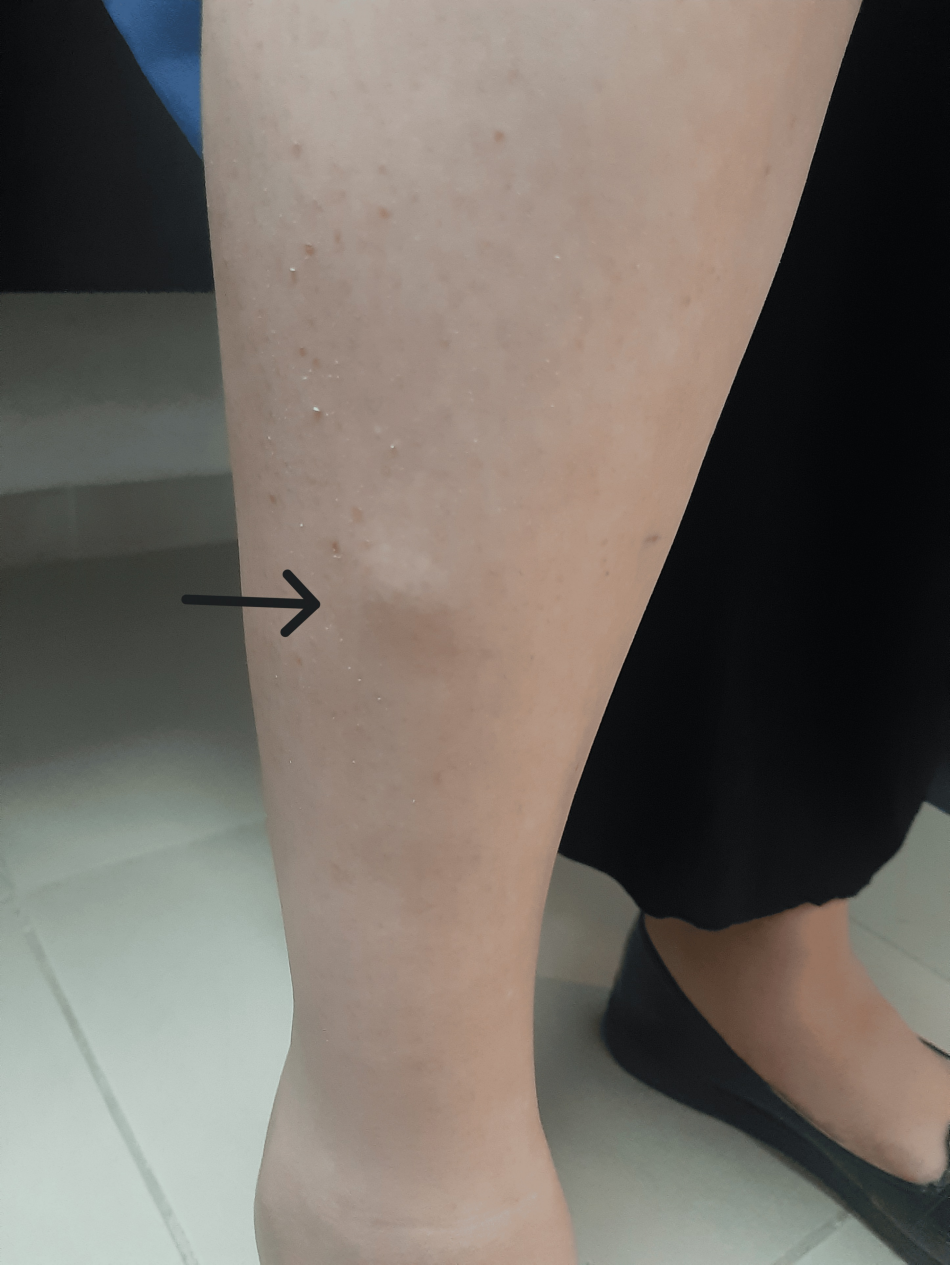
Visible protrusion of the tibialis anterior muscle on the anterolateral aspect of the middle third of the leg during weight-bearing, consistent with muscle herniation.

A 55-year-old patient with no significant past medical history presented with a tender nodule on the anterolateral aspect of the middle third of his leg, which first appeared two years ago. The nodule increased in size during prolonged standing and activity but decreased when the patient was lying down. The onset of symptoms followed a remote history of trauma from a fall down the stairs. Initially asymptomatic, the patient experienced persistent pain over the past five months, described as dull and localized with a severity rating of 7/10 on a visual analog scale, worsening with activity. There were no associated symptoms of numbness, tingling, or weakness in the lower extremities. Physical examination of the right leg revealed a soft, mildly tender, non-reducible mass measuring 1.5 cm×1.0 cm on the anterolateral aspect of the middle third of the leg (Figure 1). Sensory examination of the lower extremities was normal, with no evidence of hypoesthesia or paresthesia. Muscle strength was preserved, gait examination was unremarkable, and no limitation in ankle range of motion was observed. Peripheral pulses were intact, and the remainder of the physical examination was unremarkable bilaterally.

**Figure 2:**
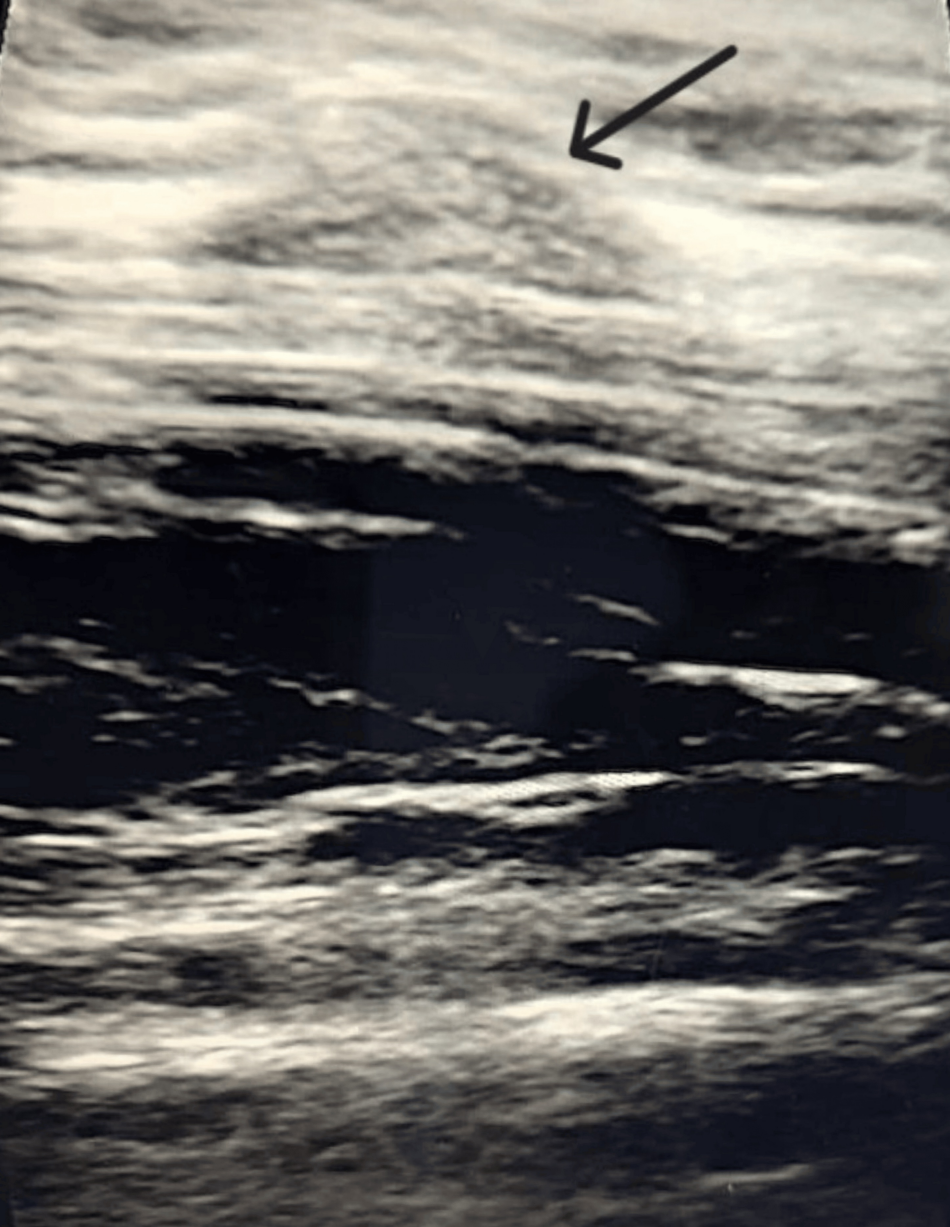
Dynamic ultrasonography demonstrating focal loss of fascial continuity with protrusion of the tibialis anterior muscle during contraction. The black arrow indicates the herniated portion of the tibialis anterior muscle protruding through the fascial defect.

Considering the patient's unremarkable medical history and examination findings, alongside the lesion's significant positional variation and lack of alarming symptoms, muscle herniation became a leading diagnostic consideration among other possible soft tissue lesions. A diagnostic ultrasound was therefore performed. Dynamic ultrasonography conducted during standing and muscle contraction demonstrated a 1 cm subcutaneous hypoechoic mass protruding through the tibialis anterior fascia, associated with a concurrent fascial defect, confirming the diagnosis of tibialis anterior muscle herniation (Figure 2).

The patient was advised to follow conservative management consisting of activity modification, relative rest, and the use of compression bandaging during daily activities. Surgical referral was considered as a secondary option in case of persistent or worsening symptoms. After three months of conservative management, the reported pain intensity decreased significantly to 1/10 on the VAS scale, and there was a slight reduction in the observed size of the muscle hernia, confirmed by clinical examination and a follow-up ultrasound. Although the patient still experienced mild exertional pain in the localized area, she expressed satisfaction with the outcome at the three-month follow-up.

## Discussion

Muscular herniation, characterized by the protrusion of muscle through its surrounding fascia, is a rare occurrence in clinical practice [1], with approximately 200 symptomatic cases of extremity muscle herniation reported in the literature [2]. However, the condition is likely underdiagnosed and underreported, particularly in asymptomatic or self-limiting cases.

The majority of these cases involve the tibialis anterior muscle [3], which is particularly susceptible to trauma due to its location and the inherent weakness of its fascia compared to other lower extremity muscles [4]. However, muscle herniation can also affect other muscles such as the extensor digitorum longus, fibularis longus, and fibularis brevis [5].

According to Ihde [6], hernias can be classified as constitutional or traumatic in origin. Constitutional hernias may stem from congenital factors or increased intracompartmental pressure induced by excessive muscular exertion and exercise [3,7], in contrast, traumatic hernias can result from penetrating trauma causing tears or punctures in the fascia, repetitive blunt trauma, overuse, or increased compartment pressure over time [3,8]. In the case discussed, a detailed interrogation of the patient revealed a history of old blunt trauma to the tibialis anterior muscle, which likely weakened the overlying fascia and eventually led to symptomatic herniation. The intermittent appearance of the swelling during weight-bearing and the absence of neurological deficits in our patient were consistent with previously reported clinical features of tibialis anterior muscle herniation.

Clinical manifestations of muscle herniation include palpable bulges, soft tissue masses, or subcutaneous nodules, which can vary in presentation from solitary to multiple and may or may not be reducible. Some cases may even present with strangulated muscle tissue [9]. The clinical diagnosis of anterior tibialis herniation relies on specific observations, including the disappearance or alteration in the size of the nodule upon manipulation of the lower extremity [10,11]. Provocative maneuvers, such as the "fencer's lunge" or forced dorsiflexion [11], can increase intracompartmental pressure, aiding in the assessment. The mass typically diminishes or resolves when the patient assumes sitting, supine, or plantar flexion positions [11]. Additionally, concerning signs may include lower extremity weakness or sensory deficits below the nodule [5,12].

While some cases, like the one reported by Nguyen et al. [5], may involve compression of nearby nerves, our patient did not exhibit signs of nerve compression. However, diagnosing anterior tibialis herniation can be challenging due to variable symptoms and a broad list of differential diagnoses, including hematomas, varicosities, angiomas, vascular malformations, epidermoid cysts, lipomas, schwannomas, tumors, muscle rupture (pseudo hernia), and central neuropathy [13].

In the past, clinical findings such as focal swelling with weight-bearing and changes in size when supine were considered sufficient for diagnosing tibialis anterior muscle herniation. However, dynamic ultrasonography is currently considered the preferred imaging modality for confirming the diagnosis because it provides accurate real-time visualization of fascial defects and muscle protrusion [11].

Evaluation via ultrasonography or MRI is essential to confirm the diagnosis, rule out other pathologies, and guide treatment decisions. While ultrasonography is operator-dependent, it is often the preferred initial imaging choice due to its ease of use and cost-effectiveness [10,14]. Dynamic imaging with ultrasound allows a real-time visualization of the hernia during limb movement and muscle contraction [10,11], aiding in the diagnosis and treatment planning [5,10,11]. However, computed tomography is less reliable in identifying fascial defects due to similar attenuation of fascia and muscle [15].

MRI is particularly useful when conservative treatment fails and surgical intervention is being considered. It offers better visualization of the musculofascial demarcation, determination of herniated muscle volume, and assists in surgical planning by assessing neighboring tissues [16].

Dynamic imaging techniques, such as those involving fast imaging with forced muscular movements like dorsiflexion and plantar flexion, improve the diagnostic accuracy of both MRI and ultrasonography, facilitating precise localization of the hernia and fascial defect [17]. Among available imaging modalities, dynamic ultrasound has been highlighted as particularly useful in confirming the diagnosis and avoiding unnecessary biopsies [11]. In our patient, dynamic ultrasonography was sufficient to confirm the diagnosis and avoid unnecessary additional investigations.

Treatment strategies for muscle herniation are controversial and depend on the severity of symptoms, ranging from conservative therapy to surgical repair. The management of tibialis anterior herniation lacks a universal approach. For asymptomatic cases, patient reassurance and education suffice [18], while most symptomatic muscle hernias are successfully treated with conservative therapy, including rest, activity restrictions and compression stockings [13,15]. Some research suggests that a more proactive approach, combining isometric, eccentric, and plyometric exercises, yields useful results [19].

In our case, conservative therapy was initially preferred because the patient did not present with neurological impairment or major functional limitation. Significant pain relief was observed after three months of conservative management, supporting this approach in selected symptomatic patients. Given the favorable clinical improvement, absence of neurological symptoms, and lack of major functional limitation, surgical intervention was not considered necessary at that stage.

If conservative treatments fail, surgery is an option. Surgical options include direct repair [20], fascial grafting [21], fasciotomy [22], partial muscular excision, and mesh grafting [23], although the optimal surgical technique remains a subject of debate.

The direct repair method involves closing the fascia defect directly by tightening the area, but it's often not feasible and may fail, leading to hernia recurrence [23]. Moreover, this technique can elevate intracompartmental pressure, especially postoperatively due to swelling, potentially causing compartment syndrome in the anterior compartment [22,24,25]. Consequently, muscle herniations, particularly in the anterior compartment of the leg, are typically not repaired through fascial closure due to the risk of subsequent compartment syndrome [24,25]. Instead, a longitudinal fasciotomy is recommended as an alternative surgical approach [22], which can effectively relieve symptoms and prevent compartment syndrome development [22,26,27].

In recent times, repair using synthetic patches has gained traction [25,26]. Marić et al. have proposed periosteal patch plasty as a viable solution, offering advantages such as accessibility, affordability, and the use of autologous material for treating tibialis anterior muscle hernias [28].

However, every surgical approach comes with drawbacks and potential complications that need careful consideration. Anatomical repair of the fascial defect (e.g., primary repair, fascial grafting, synthetic mesh) requires vigilant monitoring due to the risks of acute or chronic compartment syndrome and hernia recurrence [13,22,24,25,29]. Fascial grafting may necessitate additional or larger incisions, potentially creating new sites for hernia formation [30]. Synthetic mesh poses the risk of infection due to foreign-body implantation and may inadvertently adhere to the underlying structures [5].

## Conclusions

Clinicians should consider muscle herniation as a potential cause of chronic leg pain and intermittent soft tissue swelling, particularly when symptoms vary with posture or muscle contraction. In our patient, conservative management with activity modification and compression therapy resulted in significant pain relief and clinical improvement after three months of follow-up. Dynamic ultrasonography played an important role in confirming the diagnosis and excluding other soft-tissue lesions. This case highlights that conservative treatment may provide favorable outcomes in selected symptomatic patients and should be considered before surgical intervention.
